# Novel Intact Polar and Core Lipid Compositions in the *Pyrococcus* Model Species, *P. furiosus* and *P. yayanosii*, Reveal the Largest Lipid Diversity Amongst Thermococcales

**DOI:** 10.3390/biom10060830

**Published:** 2020-05-29

**Authors:** Maxime Tourte, Vanessa Kuentz, Philippe Schaeffer, Vincent Grossi, Anais Cario, Philippe M. Oger

**Affiliations:** 1 Univ Lyon, Univ Lyon 1, CNRS, UMR 5240, F-69622 Villeurbanne, France; maxime.tourte@univ-lyon1.fr; 2 Univ Lyon, INSA Lyon, CNRS, UMR 5240, F-69621 Villeurbanne, France; anais.cario@icmcb.cnrs.fr; 3 Univ Strasbourg, CNRS, UMR 7177 Strasbourg, France; v.kuentz@group-irsea.com (V.K.); p.schaef@unistra.fr (P.S.); 4 Univ Lyon, Univ Lyon 1, ENSL, CNRS, LGL-TPE, F-69622 Villeurbanne, France; vincent.grossi@univ-lyon1.fr

**Keywords:** archaeal membrane lipids, *Pyrococcus*, *P. furiosus*, *P. yayanosii*, phospholipids, glycerophospholipids, core lipids, tetraethers

## Abstract

Elucidating the lipidome of Archaea is essential to understand their tolerance to extreme environmental conditions. Previous characterizations of the lipid composition of *Pyrococcus* species, a model genus of hyperthermophilic archaea belonging to the Thermococcales order, led to conflicting results, which hindered the comprehension of their membrane structure and the putative adaptive role of their lipids. In an effort to clarify the lipid composition data of the *Pyrococcus* genus, we thoroughly investigated the distribution of both the core lipids (CL) and intact polar lipids (IPL) of the model *Pyrococcus furiosus* and, for the first time, of *Pyrococcus yayanosii*, the sole obligate piezophilic hyperthermophilic archaeon known to date. We showed a low diversity of IPL in the lipid extract of *P. furiosus*, which nonetheless allowed the first report of phosphatidyl inositol-based glycerol mono- and trialkyl glycerol tetraethers. With up to 13 different CL structures identified, the acid methanolysis of *Pyrococcus furiosus* revealed an unprecedented CL diversity and showed strong discrepancies with the IPL compositions reported here and in previous studies. By contrast, *P. yayanosii* displayed fewer CL structures but a much wider variety of polar heads. Our results showed severe inconsistencies between IPL and CL relative abundances. Such differences highlight the diversity and complexity of the *Pyrococcus* plasma membrane composition and demonstrate that a large part of its lipids remains uncharacterized. Reassessing the lipid composition of model archaea should lead to a better understanding of the structural diversity of their lipidome and of their physiological and adaptive functions.

## 1. Introduction

Archaea are ubiquitous in all Earth ecosystems due to peculiar physiological features that allow them to withstand environmental conditions ranging from the mildest to the harshest. One of these peculiarities lies in the structure of their cell membranes, which are composed of lipids of divergent structures and properties from the typical fatty-acyl phospho- and glyco-lipids found in Bacteria and Eukarya. The hydrophobic core of archaeal lipids is made of C_20_, C_25_ and C_40_ isoprenoid hydrocarbon chains linked to a glycerol backbone in an *sn*-2,3 configuration by ether bonds, creating cell membranes with enhanced impermeability and stability compared to that of Bacteria and Eukarya [1,2]. Archaeal lipids are classified into two groups: diethers that form membrane bilayers and membrane-spanning tetraethers that form monolayer membranes. The structural core lipid diversity includes Mono- and Dialkyl Glycerol Diethers (MGD and DGD respectively) [3], Glycerol Mono-, Di- or Trialkyl Glycerol tetraethers (GMGT, GDGT and GTGT, respectively) [4], di- and tetraethers with hydroxylated or unsaturated isoprenoid chains [5,6] and tetraethers with glycerol, butanol, pentanol and nonitol backbones [7]. The polar head group diversity of archaea mostly resembles that typical for bacterial/eukaryal lipids, with the existence of phospho- and glyco-lipids deriving from sugars [8], aminoacids [9] or combinations of both [9].

The occurrence and distribution of archaeal lipids still remain poorly characterized, partly because the classical extraction procedures may lead to the preferential extraction of some classes of lipids over others. Current data on archaeal lipids might thus not fully represent the real diversity and distribution in the original samples [10,11]. Indeed, reinvestigation of the lipid composition of several archaeal isolates using alternative procedures has almost systematically led to the discovery of novel major lipid structures. In this respect, the cases of *Methanothermobacter thermautotrophicus* [4,12,13] and *Thermococcus barophilus* [11,14] are noteworthy. Deciphering the precise and complete spectrum of archaeal lipids is now of paramount importance in order to grasp their exact biological relevance, i.e., their physiological and adaptive functions, and to explain the physico-chemical properties of the archaeal membrane under the extreme conditions Archaea most often thrive in.

*Pyrococcus furiosus* is a piezosensitive (<10 MPa), hyperthermophilic (70–113 °C, optimal 100 °C), neutrophilic (pH 5.0–9.0, optimal pH 6.8) marine (0.5–5.0% NaCl, optimal 2.0–3.0% NaCl) archaeon that was isolated from geothermally heated sediments from Vulcano Island, Italy [15]. Since it is one of the few Archaea for which a genetic system is available [16,17], *P. furiosus* is now the best-studied species from the Thermococcales order. *P. woesei*, another *Pyrococcus* species named after the father of Archaea, Carl Woese, was isolated in the same year from the same location as *P. furiosus* [18]. From the physiological and genomic points of view, both species are almost identical, as they grow optimally under the exact same conditions of temperature, salinity and pH [15,18], and exhibit 98% identity at the genome level and share most of their genomic markers [19]. However, reports of their membrane lipid compositions have shown drastic differences. Indeed, *P. woesei* was shown to produce only DGD with a phosphatidyl inositol head group [20], whereas divergent membrane lipid compositions were reported for *P. furiosus* [21,22]. Sprott et al. first identified ether lipids with the typical DGD and GDGT hydrophobic cores associated with diverse polar head groups, including both phospho- and glyco-lipids [21]. However, more recently, Lobasso et al. identified novel core lipid structures in *P. furiosus*, namely GDGT with two cyclopentane rings and an ether lipid cardiolipin [22]. Additionally, the core lipid compositions of *P. furiosus* and *P. woesei* differ greatly from that of other *Pyrococcus* members, such as *P. abyssi*, which synthesizes 15% of DGD and 85% of GDGT, including ca. 9% with two cyclopentane rings [23], *P. horikoshii*, which mostly produces tetraether lipids (>90%) with more than 15% in the GMGT monoalkyl form [24,25,26], and *P. kukulkanii*, which seemingly produces only diether lipids [27].

In an effort to further explore the membrane lipid composition in the *Pyrococcus* genus, we here rigorously assayed the intact polar lipid (IPL) and core lipid (CL) compositions of two emblematic species of this genus, *P. furiosus*, the first isolated and best characterized *Pyrococcus* species [15], and *P. yayanosii*, its most piezophilic member, whose lipids are yet unknown [28]. In addition, as it is now well established that archaeal lipid extractions and analyses can be biased [10,11], we used *Thermococcus kodakarensis*, for which the most complete IPL and CL compositions have been described [29], as a quality control for our lipid extraction procedure and analysis. We show that *P. yayanosii* synthesizes phosphatidylinositol-based IPL with typical DGD and GDGT cores, while several novel IPL and CL could be identified in *P. furiosus*, notably GMGT and GDGT with up to four cyclopentane rings.

## 2. Materials and Methods

### 2.1. Microorganisms and Growth Conditions

*Pyrococcus furiosus* strain DSM3638 was isolated from geothermally heated sediments from the beach of Porto di Levante, Vulcano Island, Italy [15]. *Thermococcus kodakarensis* strain KOD1 was isolated from a solfatara in a wharf of Kodakara Island, Kagoshima, Japan [30]. Both strains were purchased from the Deutsche Sammlung für Mikroorganismen und Zellkulturen (DSMZ), Braunschweig, Germany. *Pyrococcus yayanosii* strain CH1 was isolated from the 4100 m deep Ashadze hydrothermal vent, on the mid-Atlantic ridge [28]. *P. yayanosii* was obtained from the UBOCC (Université de Bretagne Occidentale-Type Culture Collection, Brest, France). Cultures were grown under strict anaerobiosis in a rich medium established for Thermococcales [28], containing 3% NaCl and 10 g·L^−1^ elemental sulfur, at pH 6.8. *P. furiosus* and *T. kodakarensis* were cultivated at atmospheric pressure and 98 and 85 °C, respectively, whereas *P. yayanosii* was grown at 52 MPa and 98 °C. The medium was reduced by adding Na_2_S (0.1% *w/v* final) before inoculation. Growth was monitored by counting with a Thoma cell (depth 0.01 mm) using a light microscope (life technologies EVOS^®^ XL Core 400×, Waltham, MA, USA).

Cells of 250-mL cultures in late exponential phase were recovered by centrifugation (4000× *g*, 45 min, 4 °C) and rinsed twice with an isotonic saline solution (3% *w/v* NaCl). Cultivation under high hydrostatic pressure being much more demanding, a significantly lower biomass was recovered for *P. yayanosii* than for *P. furiosus* and *T. kodakarensis*. The cell pellets were lyophilized overnight and kept at −80 °C until lipid extraction.

### 2.2. IPL Extraction and HPLC-ESI-MS Analysis

**Figure 1:**
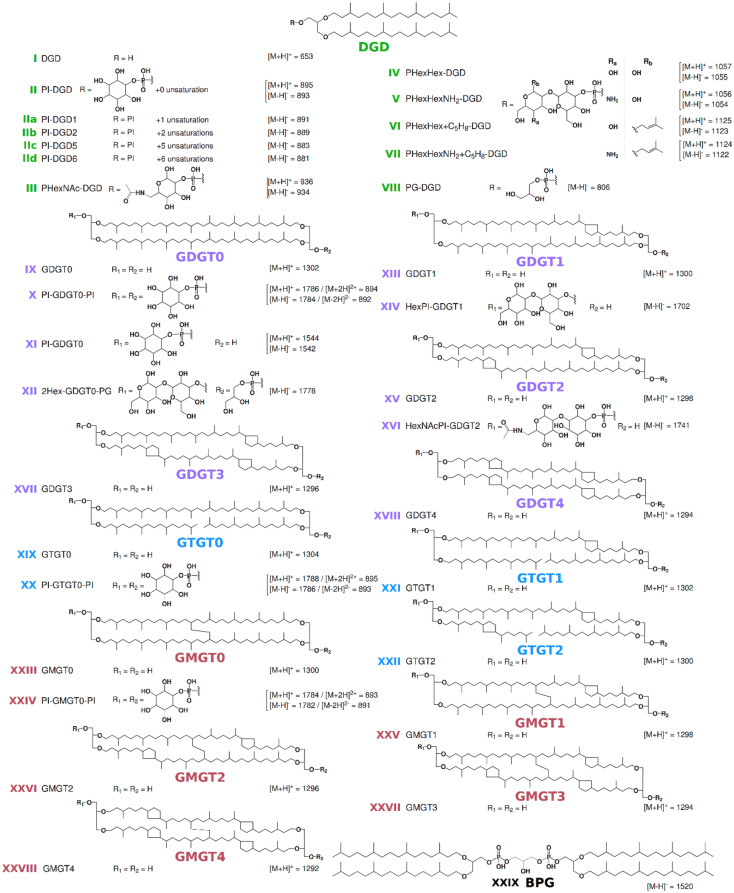
Core and intact polar lipids identified in *Pyrococcus furiosus*, *Pyrococcus yayanosii* and *Thermococcus kodakarensis* and other lipids mentioned in the text. Shorthand nomenclature is indicated. The protonated and/or deprotonated mass–charge ratios are indicated for each structure when detected. Ammoniated and sodiated adducts were also detected and used for integration, but are not represented here. Core structures: dialkyl glycerol diethers (DGD; green) with 0 (**I**, **II**, **III**, **IV**, **V**, **VI**, **VII** and **VIII**) and with 1 (**IIa**), 2 (**IIb**), 5 (**IIc**) and 6 (**IId**) unsaturations; glycerol dialkyl glycerol tetraethers (GDGT; purple) with 0 (**IX**, **X**, **XI** and **XII**) and with 1 (**XIII** and **XIV**), 2 (**XV** and **XVI**), 3 (**XVII**) and 4 cyclopentane rings (**XVIII**); glycerol trialkyl glycerol tetraethers (GTGT; blue) with 0 (**XIX** and **XX**) and with 1 and 2 cyclopentane rings (**XXI** and **XXII**); glycerol monoalkyl glycerol tetraethers (GMGT, red) with 0 (**XXIII** and **XXIV**) and with 1 to 4 cyclopentane rings (**XXV** to **XXVIII**). Polar head groups: phosphatidyl inositol (PI, **II**, **IIa** to **IId**, **X**, **XI**, **XIV**, **XVI**, **XX** and **XXIV**), (phosphatidyl) N-acetylhexoseamine ((P)HexNAc, **III** and **XVI**), glycosylated phosphatidyl hexose (PHexHex, **IV** and **XIV**), PHexHex bearing an additional mass of 68 (PHexHex+C_5_H_8_, **VI**), ammoniated PHexHex (PHexHexNH_2_, **V**), PHexHexNH_2_ bearing an additional mass of 68 (PHexHexNH_2_+C_5_H_8_, **X**), phosphatidyl glycerol (PG, **VIII** and **XII**), dihexose (2Hex, **XII**), PI bearing an additional HexNAc head group (HexNAcPI, **XIV**). In addition, the peculiar structure of an ether cardiolipin gathering two DGD linked by a bisphosphatidyl glycerol (BPG, **XXIX**) is represented. Positions of cyclopentane rings, covalent bound between the two alkyl chains and additional groups on the polar heads are drawn arbitrarily in this Figure.

IPL were extracted using a modified Bligh and Dyer (B&D) method [31]. Briefly, dried cells were extracted with a monophasic mixture of methanol/dichloromethane/purified water (MeOH/DCM/H_2_O; 1:2.6:0.16; *v/v/v*) using a sonication probe for 15 min. After centrifugation (2500× *g*, 5 min), the supernatant was collected and the extraction procedure was repeated twice. The supernatants were pooled, dried under reduced pressure and solubilized in Methanol/dichloromethane (DCM) (1:5; *v/v*). A significant amount of sulfur from the growth medium was extracted alongside archaeal lipids, and the total lipid dry mass of the three Thermococcales species was thus not estimated. IPL were analyzed using high-performance liquid chromatography coupled with electrospray ionization mass spectrometry (HPLC-ESI-MS) using an HP 1100 series HPLC instrument equipped with an auto-injector and a Chemstation chromatography manager software. Separation was achieved on a Diol 5 µm column (250 mm × 2.1 mm, Inertsil^®^, GL Science, Torrance, CA, USA) maintained at 30 °C. The injection volume was set to 5 µL. Di- and tetraether IPL were eluted in the same run with a flow rate of 0.2 mL·min^−1^, using the nonlinear gradient described by Sinninghe Damsté et al. [32]. Detection was achieved using an Esquire 3000+ ion trap mass spectrometer with an electrospray ionization source in positive and negative modes. The conditions for the MS analyses were as follows: nebulizer pressure 30 psi, cone tension 40 V, drying gas (N_2_) flow 8 L·min^−1^ and temperature 340 °C, capillary voltage 5 kV (negative mode) and −4 kV (positive mode), mass range *m/z* 650–2000. IPL structures were determined using Bruker Data Analysis software by comparing the obtained protonated molecular ion masses (and their NH_4_^+^ and Na^+^ adducts) and specific fragmentation patterns with previously described ones [22,29]. Identified structures and their respective protonated (positive mode) and deprotonated (negative mode) molecular ion masses are displayed in Figure 1. For each archaeal species, IPL relative abundances were determined by integration of the peak area on the mass chromatograms corresponding to the protonated, ammoniated and sodiated adducts in positive mode and to the deprotonated adduct in negative mode. As no archaeal GDGT-based phosphoglycolipid was commercially available, no relevant standard could be used to assay the response factors of the detected IPL. Although different response factors are to be expected in ESI-MS (ElectroSpray Ionizatio-Mass Spectroscopy), notably for lipids bearing distinct classes of polar head groups, a response factor of 1 was arbitrarily chosen for comparison of the relative IPL abundances between the three species investigated.

### 2.3. CL Preparation and HPLC-APCI-MS Analysis

In order to exhaustively analyze the CL composition of the strains, polar head groups were removed using acid methanolysis (1.2 N HCl in MeOH at 110 °C for 3 h) of (1) the total lipid extracts, (2) the cell residue obtained after lipid extraction and (3) the intact cell pellets (26). Because of the low biomass of *P. yayanosii*, the CL preparation was conducted only on the total lipid extract and the cell residue for this species. The hydrolyzed lipids were extracted using MeOH/DCM (1:1, *v/v*; ×3) and filtrated over celite. The solvent of the resulting CL extracts was removed under reduced pressure, and the extracts were resolubilized in *n*-hexane/isopropanol (99:1, *v/v*). CL analysis was conducted using HPLC coupled with atmospheric-pressure chemical ionization MS (HPLC-APCI-MS) as described in Tourte et al. [26]. Under our analytical conditions, DGD was the only form of diether CL detected, whereas numerous tetraether structures were identified, namely GDGT, GTGT and GMGT and their cyclopentane ring-containing derivatives. A chemically synthesized core DGD and a core GDGT with no cyclopentane ring (GDGT0) isolated from a culture of *M. thermautotrophicus* were used to determine the response factors of the different CL under our analytical conditions. The analysis of a 2/1 standard solution (DGD/GDGT0, % mol) led to a molar response factor of DGD that was ca. 10 times lower than that of GDGT0. Similarly, to what is frequently found in the literature, a response factor of 1 was assumed between GDGT0 and the other tetraethers. For each archaeal species, CL relative abundances were determined by integration of the peak area on the mass chromatograms corresponding to the protonated adduct only, and the relative abundance of DGD was corrected by a factor of 10 relative to that of tetraethers.

## 3. Results

**Figure 2:**
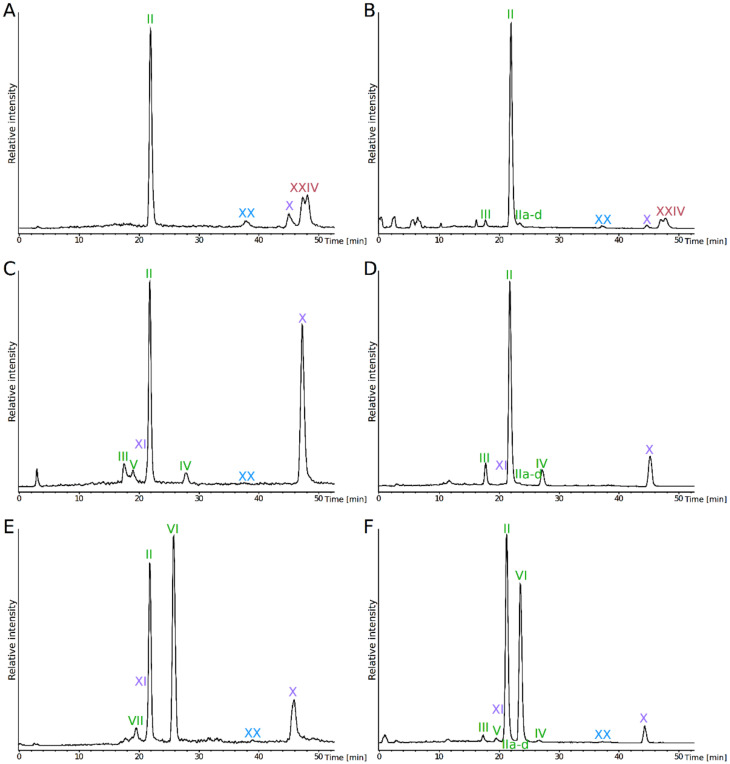
HPLC-ESI-MS chromatograms of intact polar lipids of *Pyrococcus furiosus* (**A**, **B**), *P. yayanosii* (**C**, **D**) and *Thermococcus kodakarensis* (**E**, **F**). Intact polar lipids were detected in positive (**A**, **C** and **E**) and negative (**B**, **D** and **F**) ion modes. Chromatograms were drawn by extracting the following protonated and deprotonated ions: positive mode: 893, 894, 895, 936, 1056, 1057, 1124, 1125, 1544, 1784, 1786 and 1788; negative mode: 881, 883, 889, 891, 892, 893, 934, 1054, 1055, 1122, 1123, 1542, 1782, 1784, and 1786. Refer to Figure 1 for lipid structures and their corresponding masses.

**Figure 3:**
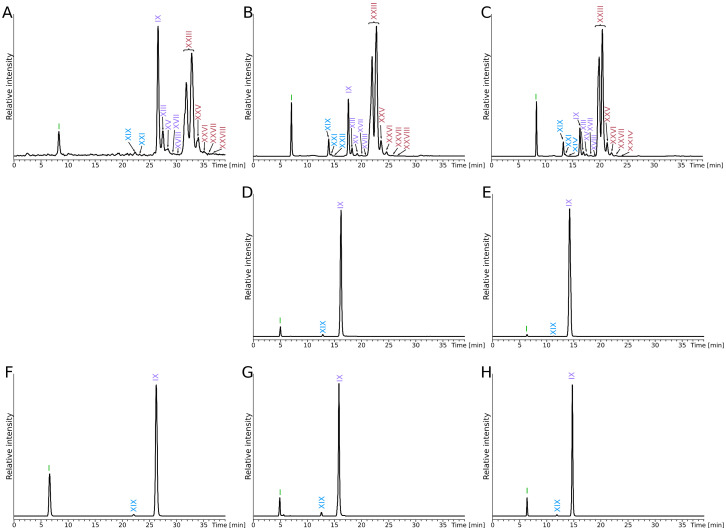
HPLC-APCI-MS chromatograms of core lipids from *P. furiosus* (**A**–**C**), *P. yayanosii* (**D**, **E**) and *Thermococcus kodakarensis* (**F**–**H**). Total CL (**A** and **F**) were recovered after direct methanolysis of the cell pellets. Note that the methanolysis of *P. yayanosii* biomass was not performed due to the low amounts of material available for this species. CL from the intact polar lipids (**B**, **D** and **G**) were obtained after methanolysis of the intact polar lipids (IPL) extracts. Residual CL (**C**, **E** and **H**) were recovered after methanolysis of the pre-extracted cells. Chromatograms were drawn by extracting the following protonated ions: 653, 1292, 1294, 1296, 1298, 1300, 1302 and 1304. Refer to Figure 1 for lipid structures and their corresponding masses.

The analysis of the total lipid extracts from *Pyrococcus furiosus*, *P. yayanosii* and *Thermococcus kodakarensis* obtained with our modified B&D extraction procedure showed various IPL structures (roman numbers hereafter refer to the structures presented in Figure 1) and distinct lipid compositions in these three closely related hyperthermophilic archaea (Figure 2). *P. furiosus* IPL were strongly dominated by a DGD with a phosphatidyl inositol polar head group (PI-DGD, compound **II**, [M-H]^-^ 893; ca. 59% and 84% in positive and negative ion modes, respectively; Figure 2A,B and Table 1). Minute amounts of putative derivatives of PI-DGD II were further observed in negative mode. The deprotonated molecular ions detected ([M-H]^-^ 891, 889, 883 and 881) could correspond to homologues of PI-DGD II with 1, 2, 5 and 6 unsaturations (compounds IIa to IId), respectively, but the absence of clear fragmentation patterns however impeded their unambiguous identification. Three additional compounds (compounds X, XX and XXIV) with different retention times and masses (1788, 1786 and 1784, respectively) likely corresponding to tetraethers, were also detected on the HPLC-ESI-MS chromatogram of *P. furiosus*. With a predominant [M+H]^+^ ion at *m/z* 1786, compound X was identified as PI-GDGT0-PI. The two other compounds (XX and XXIV) exhibited [M+H]^+^ ions shifted respectively upwards and downwards by two mass units. The hydrolysis experiments carried out on the solvent extract, the pre-extracted cells and the whole biomass of *P. furiosus* systematically yielded CL dominated by GTGT0 XIX, GDGT0 IX and GMGT0 XXIII (Figure 3A–C). Compounds XX and XXIV were thus tentatively identified as PI-GTGT0-PI and PI-GMGT0-PI, respectively. Confusion with ring-containing homologues was not possible due to the well-differentiated retention times observed between acyclic and cyclic tetraether CL (Figure 3A–C). Interestingly, compound XXIV appeared as a doublet of peaks likely corresponding to isomers (Figure 2), as did GMGT0 XXIII released by methanolysis (Figure 3A–C), which further supported the PI-GMGT0-PI structure proposed for compound XXIV. Assuming a response factor of 1 in HPLC-ESI-MS between the different tetraether IPL, PI-GMGT0-PI XXIV was the most abundant tetraether lipid in our modified B&D extract from *P. furiosus*, representing ca. 28% and 11% of the total IPL in positive and negative ion modes, respectively, whereas PI-GDGT0-PI X and PI-GTGT-PI XX only reached ca. 8% and 2% and ca. 5% and 1% of the total IPL, respectively (Figure 2A,B and Table 1). In addition, small amounts of DGD with a phosphatidyl N-acetylhexosamine polar head group (PHexNAc-DGD; compound III, ca. 2% in negative mode) were detected in *P. furiosus* (Figure 2A,B and Table 1).

Similarly to *P. furiosus, P. yayanosii* IPL were dominated by PI-DGD II (ca. 38% and 61% in positive and negative ion modes, respectively), together with unsaturated PI-DGD IIa to IId (trace amounts) and PHexNAc-DGD III (ca. 7% and 10%) (Figure 2A,C and Table 1). *P. yayanosii* tetraether lipids were composed of PI-GDGT0-PI X (ca. 44% and 22%), with minute amounts of PI-GTGT0-PI XX. A greater diversity of diether-based IPL was observed however in *P. yayanosii* compared to *P. furiosus*, including DGD with a glycosylated phosphatidyl hexose polar head group (PHexHex-DGD, compound IV, ca. 4% and 7%) and an uncharacterized DGD (compound VI, ca. 5% in positive ion mode). The latter compound showed a [M+H]^+^ ion at *m/z* 1125, which corresponds to that of PHexHex-DGD ([M+H]^+^ 1057) shifted upwards by 68 mass units. Although no fragmentation pattern was available for this compound VI, we suggest that it could be a derivative of PHexHex-DGD bearing an additional isoprene unit attached to the polar head (PHexHex+C_5_H_8_-DGD VI; Figure 1 and Figure 2C,D). In contrast to *P. furiosus, P. yayanosii* did not exhibit PI-GMGT0-PI but another tetraether-based IPL, which could be identified as PI-GDGT0 XI (ca. 3% and trace amounts in positive and negative ion modes, respectively).

*Thermococcus kodakarensis* exhibited the greatest diversity of DGD-based IPL of the three Thermococcales species investigated. It consisted of the same diethers as those from *P. yayanosii*, with the addition of a derivative of PHexHex-DGD IV ([M+H]^+^ 1057), for which one of the hexose group is replaced by a hexosamine moiety (PHexHexNH_2_-DGD V, [M+H]^+^ 1056), and an uncharacterized DGD (compound VII; Figure 2E,F). Similarly to PHexHex+C_5_H_8_-DGD VI, the protonated molecular ion of compound VII ([M+H]^+^ 1124) corresponds to that of PHexHexNH_2_-DGD ([M+H]^+^ 1056) shifted upwards by 68 mass units. Compound VII was thus assumed to be a derivative of PHexHexNH_2_-DGD bearing an additional isoprene unit attached to the polar head (PHexHexNH_2_+C_5_H_8_-DGD VII; Figure 1 and Figure 2E,F). In contrast to both *P. furiosus* and *P.* *yayanosii*, whose IPL were dominated by PI-DGD II, the most abundant IPL of *T. kodakarensis* was PHexHexNH_2_-DGD V (ca. 41% and 42%), although high amounts of PI-DGD II (ca. 31% and 51%) were also recovered (Figure 2E,F and Table 1). PI-GDGT0-PI X and PI-GDGT0 XI represented respectively ca. 14% and 9% of *T. kodakarensis* IPL in the positive ion mode and ca. 3% and 2% in the negative ion mode, respectively (Table 1).

In agreement with its IPL composition, the CL recovered from *P. furiosus* showed a large diversity of structures, which included DGD I, GTGT0 XIX, GDGT0 IX and GMGT0 XXIII (Figure 3A–C). However, a variety of cyclopentane ring-containing tetraethers, including GTGT with 1 and 2 rings (GTGT1 and 2, XXI and XXII, respectively), GDGT with 1 to 4 rings (GDGT1 to 4, XIII, XV, XVII and XVIII, respectively) and GMGT with 1 to 4 rings (GMGT1 to 4, XXV to XXIIIV, respectively), was obtained upon acidic methanolysis of *P. furiosus* lipid extract, pre-extracted cells and biomass (Figure 3A–C). Correcting for the response factor for diethers relative to tetraethers [26], we determined that the most abundant CL in the three different methanolysates of *P. furiosus* were DGD I (ca. 44%, 45% and 36%, respectively) and GMGT0 XXIII (ca. 41%, 43% and 33%, respectively) (Table 2). GDGT0 IX was present in relatively high abundance only in the methanolysate of the intact biomass (ca. 17%), whereas methanolysis of the lipid extract and of the pre-extracted cells both yielded rather low proportions of it (ca. 6% and 4%, respectively). Although GTGT and ring-containing tetraethers were detected in the three CL extracts, they were systematically recovered in low abundances, i.e., below 5% (Table 2). In contrast to *P. furiosus*, the CL recovered from *P. yayanosii* and *T. kodakarensis* exhibited a low diversity of core structures, consisting exclusively of DGD I, GTGT0 XIX and GDGT0 IXV (Figure 3D–H). GDGT0 IX was the most abundant CL in *P. yayanosii* extracts, reaching ca. 64% and 92% in the methanolysates of the IPL and of the pre-extracted cells, respectively (Table 2). Methanolysis of the biomass of *T. kodakarensis* yielded more diethers than tetraethers, i.e., ca. 56% and 43%, respectively, whereas that of its IPL and pre-extracted cells released a slight majority of tetraethers (ca. 61% and 50%, respectively; Table 2).

## 4. Discussion

Our study reports the IPL and CL compositions of *Pyrococcus furiosus, Thermococcus kodakarensis* and, for the first time, that of the obligate piezophile *P. yayanosii*. Since the IPL composition of *T. kodakarensis* was recently reported by Meador et al. [29], who coupled a B&D with trichloroacetic acid extraction with HPLC-ESI-MS analysis, this species represented a control of our lipid extraction procedure and membrane lipid analysis. Under our analytical conditions, *T. kodakarensis* exhibited the most diverse IPL composition of the three species analyzed, i.e., PI-DGD II, PHexNAc-DGD III, PHexHex-DGD IV, PHexHexNH_2_-DGD V, PHexHex+C_5_H_8_-DGD VI and PHexHexNH_2_+C_5_H_8_-DGD VII, in addition to the tetraether-based IPL PI-GTGT0-PI XX, PI-GDGT0-PI X and PI-GDGT0 XI (Figure 2E,F). These IPL represented the major IPL previously recovered by Meador et al. [29] using comparable extraction and analytical methods and under similar growth conditions. Only the minor IPL detected by Meador et al. [29], i.e., glycolipids, aminoacid-based and phosphatidylglycerol(PG)-based lipids, altogether representing less than 10% of *T. kodakarensis* IPL, were not detected here. Thus, our extraction and analytical procedures prove reliable in describing the dominant IPL of Thermococcales species.

The IPL composition of the obligate piezophilic archaeon *P. yayanosii* consisted of PI-DGD II, PHexNAc-DGD III, PHexHex-DGD IV, PHexHexPI+C_5_H_8_-DGD VI, PI-GTGT0-PI XX, PI-GDGT0-PI X and PI-GDGT0 XI (Figure 1 and Figure 2C,D). In comparison, *P. furiosus* displayed a lower diversity of polar head groups, i.e., PI and PHexNAc, but attached to a much wider variety of CL, i.e., DGD, GDGT0, GTGT0 and GMGT0 (Figure 1 and Figure 2A,B). Under our analytical conditions, *P. yayanosii* did not exhibit specific IPL compared to its piezosensitive relatives. Our results thus suggest that the adaptation to high hydrostatic pressures does not involve specific core structure, but might instead be supported by particular polar head groups that are not detected by our procedure, or by other molecules such as apolar polyisoprenoids as proposed by Cario et al. [11].

Only five IPL, i.e., PI-DGD II, P-HexNAc III, PI-GTGT0-PI XX, PI-GDGT0-PI X and PI-GMGT0-PI XXIV (Figure 1 and Figure 2A,B), were unambiguously detected in our *P. furiosus* lipid extract. This IPL composition contrasts with those previously reported for this species, although PI-DGD II was systematically the most abundant IPL detected [21,22]. GMGT-based CL [4,25,26] and IPL [33] have been identified in numerous archaea, including some Thermococcales but, to the best of our knowledge, our study constitutes the first identification of GMGT-based lipids in *P. furiosus* and the first report of PI-GMGT0-PI XXIV and PI-GTGT0-PI XX as archaeal IPL. By contrast, Sprott et al. [21] tentatively identified PG-based IPL, such as PG-DGD VIII and dihexose(2Hex)-GDGT0-PG XII (Figure 1), in addition to PI-DGD II and PHexNAc-DGD III. They also reported the presence of PI-GDGT0 XI, a compound that we did identify in *P. yayanosii* and *T. kodakarensis* but not in *P. furiosus* (Figure 2). Such divergence in the detection of PI-GDGT0 XI between studies suggests either that it is a biosynthetic intermediate between core GDGT0 IX and PI-based tetraethers, or that it is a partial hydrolysis product of other IPL, such as PI-GDGT0-PI X. More recently, Lobasso et al. [22] reported saturated and unsaturated PI-DGD II and IId, PHexNAc-DGD III and PI-GDGT0-PI X, and tentatively identified ring-containing GDGT-based IPL, i.e., HexPI-GDGT1 XIV and HexNAcPI-GDGT2 XVI (Figure 1) in *P. furiosus*. This study also reported for the first time the presence of the diphytanyl glycerol analogue of cardiolipin XXIX in a hyperthermophilic archaeon (Figure 1), whereas this compound is regularly detected in halophilic archaea [34]. The two previous and the present study of *P. furiosus* membrane lipids thus highlight a large variety of its lipid membrane composition, and place *P. furiosus* as a prime model for future elucidation of archaeal lipid structural and adaptive functions.

The comparison of these three studies also shows that diverging results can be obtained when analyzing the IPL composition of the same species, and several scenarios might be invoked to explain such discrepancies. First, the lipid compositions of closely related strains might greatly diverge from one another. For instance, while the strain used in our study and that of Lobasso et al. [22] was obtained from the DSMZ (though certainly at very different time periods), that of Sprott et al. [21] was provided by one of their colleagues. It is thus likely that the three strains used have diverged, including from the lipid point of view. Second, cultures of 0.10, 0.25 and 300 L were employed in Sprott et al. [21], in the present study and in Lobasso et al. [22], respectively, which would potentially only allow to detect the most abundant IPL in the present study and that of Sprott et al. [21] while the bigger culture volumes in the study of Lobasso [22] allowed to access rarer lipids, such as HexPI-GDGT1 XIV and HexNAcPI-GDGT2 XVI. In our extracts, PHexHex and PHexNAc polar head groups were only observed attached to DGD and not to GDGT core structures (Figure 2A,B), and *P. furiosus* displayed low proportions of PHexNAc-DGD III only (2% in negative ion mode; Table 1). In parallel, cyclopentane ring-containing GDGT1 XIII and GDGT2 XV were also detected in low proportions among the CL released from intact cells (ca. 4% and 1%, respectively; Figure 3A–C and Table 2). Such minute amounts of both polar head groups and core structures probably prevented minor IPL, such as XIV and XVI, from being detected in our study. Third, *T. kodakarensis* was demonstrated to adapt its membrane lipid distribution to its growth stage and to the medium composition [29]. The growth temperature and medium composition as well as the incubation time employed in our study greatly differed from those used by Sprott et al. [21] and Lobasso et al. [22], and this probably also contributed to the distinct lipid composition unveiled by the different studies. Fourth, it has been shown that even slight changes in the extraction procedure might lead to distinct lipid recovery rates and thus yield IPL fractions with different compositions [10]. Our study (B&D extraction with a higher proportion of DCM), and those of Sprott et al. [21] (IPL precipitation after classic B&D extraction) and Lobasso et al. [22] (classic B&D extraction) employed quite comparable extraction procedures but recovered drastically different IPL compositions from *P. furiosus*. Fifth, it has been determined that, similarly to extraction procedures, different analytical techniques may induce the preferential detection of some specific classes of lipids over others, e.g., the negative ionization mode enhances the response of PG-based IPL ions compared to the positive mode [35] while matrix-assisted laser desorption/ionization–time of flight (MALDI-TOF) preferentially spots lipids with net charges or high proton affinity [36]. While HPLC-ESI-MS was employed in our study, Lobasso et al. [22] and Sprott et al. [21] investigated total lipid extracts using MALDI-TOF-MS without prior HPLC lipid separation and fast atom bombardment (FAB)-MS, respectively, which potentially also contributed to the lipid composition discrepancies observed between studies. Altogether, these observations illustrate the plethora of parameters that can influence the lipid diversity recovered from pure cultures of a single archaeal species, and argue in favor of extreme cautiousness when dealing and analyzing archaeal lipid data. Similarly to *P. furiosus*, further reevaluations of the lipid diversity of previously analyzed archaea might help constraining the variability across studies and refine our knowledge of the archaeal lipidome.

To further evaluate our extraction and analytical procedures and determine whether they were able to reveal the majority of IPL from *P. furiosus*, *P. yayanosii* and *T. kodakarensis*, acidic methanolysis was performed on the cell pellets (Figure 3A,F), the B&D extracts (Figure 3B,D,G) and the extracted cells (Figure 3C,E,H). First, the methanolysates from residual cells after B&D extraction revealed the presence of remaining, non-extracted lipids (Figure 3C,E,H and Table 1), showing that the B&D extraction procedure does not lead to the complete recovery of the lipid content of Thermococcales cells, as already demonstrated in previous studies [10,11]. The methanolysates of intact cells of the three species predominantly contained tetraethers, which represented from ca. 50% up to 93% of the total CL (Table 1). By contrast, tetraether-based IPL represented from ca. 5% to 47% of the lipid extracts of the three strains (Table 2). Such large discrepancies in di- and tetraether distributions between extracted IPL and CL released upon hydrolysis of intact cells may indicate that tetraethers are much more reluctant to extraction by the B&D procedure than their diether counterparts. The elevated proportions of DGD in the methanolysates of total lipid extracts, ranging from ca. 36% to 56% (Table 1), further support this assumption. In addition, the lipid extracts from *P. furiosus*, *P. yayanosii* and *T. kodakarensis* were vastly dominated by PI-based IPL, which represented from ca. 58% to ca. 98% of *T. kodakarensis* and *P. furiosus* lipid extracts in negative ion mode, respectively (Figure 2 and Table 1). It is very unlikely that PI-based phospholipids are the only ones present in these strains, because membranes of divergent lipid compositions are essential in cells to host specific membrane functionalities. Furthermore, we could obtain a *P. furiosus* mutant in which the genes involved in the specific fixation of the PI polar headgroup and derivatives was knocked out, showing that the strain can be viable in the absence of lipids of this class. Thus, in addition to DGD-based IPL, our extraction procedure and LC–MS analysis seem to strongly favor PI-based IPL. We also noted a great inefficiency to fully extract tetraether-based lipids and some diethers, suggesting that the unextracted IPL may harbor polar head groups reluctant to the current extraction methods. Based on the existence of ether-based cardiolipin [22] and covalently bonded lipids to membrane proteins in Archaea [35], such undetected IPL might for instance be involved in intricate lipid–lipid or lipid–protein complexes that are out of our analytical window.

## 5. Conclusions

We reassessed the intact and core lipid compositions of *P. furiosus* and unraveled the presence of 19 membrane lipids in this archaeon, including a variety of core structures, i.e., DGD, GMGT, GDGT, GTGT, and a few polar head groups, i.e., PI and PHexNAc. Combined with the previous studies of Sprott et al. [21] and Lobasso et al. [22], this brings the number of known lipid structures in *P. furiosus* to at least 25. The CL released upon acid methanolysis of extracted cells suggest that this number might be even greater, and that a vast diversity of *P. furiosus* IPL remains elusive because of difficulties of extraction and/or analysis. Although *P. furiosus* lipids have been investigated multiple times before, our data further demonstrate that extraction and analytical conditions are of paramount importance and require a great deal of rigor and some more improvements to access and comprehend the complete archaeal lipidome. Altogether, these results illustrate the complexity and uniqueness of the *P. furiosus* membrane structure and promote this species as a prime model of Thermococcales to elucidate archaeal lipid diversity and functions. The first membrane lipid characterization of *P. yayanosii*, the most piezophilic Thermococcales member isolated so far, showed a relatively low diversity of CL bearing exclusively phosphosugars and derivatives as polar heads. Our results bring the number of Thermococcales with known IPL diversity to eight, five of which belong to the *Pyrococcus* genus. The lipid diversity reported here contrasts with the much simpler lipid composition of closely related species, such as *P. woesei*, which only exhibited PI-DGD [20], and argue in favor of further in-depth (re)investigations of the Thermococcales lipidome to better characterize their lipid diversity and related physiological and adaptive functions.

## Figures and Tables

**Table 1:** Intact polar lipids (IPL) composition (relative %) of *Pyrococcus furiosus*, *Pyrococcus yayanosii* and *Thermococcus kodakarensis*.

Species	MS Mode	Diethers *						Tetraethers *			
		DGDsat+uns	DGD					GDGT0		GTGT0	GMGT0
		PI	PHexNAc	PHexHex	PHexHexNH_2_	PHexHex+C_5_H_8_	PHexHexNH_2_+C_5_H_8_	PI+PI	PI	PI+PI	PI+PI
		II+IIa+IIb+IIc+IId	III	IV	V	VI	VII	IX	X	XX	XXIV
*P. furiosus*	ESI+	59	ND	ND	ND	ND	ND	8	ND	5	28
	ESI-	84	2	ND	ND	ND	ND	2	ND	1	11
*P. yayanosii*	ESI+	38	7	4	ND	5	ND	44	3	Traces	ND
	ESI-	61	9	7	ND	ND	ND	22	Traces	ND	ND
*T. kodakarensis*	ESI+	31	ND	ND	41	ND	5	14	9	Traces	ND
	ESI-	51	1	Traces	42	Traces	ND	3	2	Traces	ND

* Relative proportions account for protonated, ammoniated and sodiated adducts in positive mode and for deprotonated adducts in negative mode, and were calculated assuming a response factor of 1 for all IPL (see methods). Traces, <1%. ND: not detected.

**Table 2:** Core lipid composition (relative %) of the total cell pellet (totCL), the total lipid extract (CLfromIPL) and the pre-extracted pellet (resCL) of *Pyrococcus furiosus*, *P. yayanosii* and *Thermococcus kodakarensis*.

Species	Extract	Diether *	Tetraethers *												
		DGD	GDGT0	GDGT1	GDGT2	GDGT3	GDGT4	GTGT0	GTGT1	GTGT2	GMGT0	GMGT1	GMGT2	GMGT3	GMGT4
		I	IX	XIII	XV	XVII	XVIII	XIX	XXI	XXII	XXIII	XXV	XXVI	XXVII	XXVIII
*P. furiosus*	totCL	36	17	4	1	Traces	Traces	Traces	Traces	Traces	33	6	1	Traces	Traces
	CLfromIPL	44	6	Traces	Traces	Traces	Traces	2	Traces	Traces	41	4	Traces	Traces	Traces
	resCL	45	4	Traces	Traces	Traces	Traces	2	Traces	Traces	43	4	Traces	Traces	Traces
*P. yayanosii*	CLfromIPL	35	64	ND	ND	ND	ND	Traces	ND	ND	ND	ND	ND	ND	ND
	resCL	7	92	ND	ND	ND	ND	Traces	ND	ND	ND	ND	ND	ND	ND
*T. kodakarensis*	totCL	77	23	ND	ND	ND	ND	Traces	ND	ND	ND	ND	ND	ND	ND
	CLfromIPL	56	43	ND	ND	ND	ND	1	ND	ND	ND	ND	ND	ND	ND
	resCL	50	49	ND	ND	ND	ND	Traces	ND	ND	ND	ND	ND	ND	ND

* Relative proportions account for protonated adducts only and were calculated using a response factor of 1/10 for DGD I relative to tetraether lipids (see methods). Traces, <1%. ND: not detected.

## Abbreviations

IPL	Intact polar lipid(s)
CL	Core lipid(s)
MeOH	Methanol
DCM	Dichloromethane
HPLC	High-performance liquid chromatography
MS	Mass spectrometry
ESI	Electrospray ionization
APCI	Atmospheric-pressure chemical ionization
MGD	Monoalkyl glycerol diethers
DGD	Dialkyl glycerol diethers
GDGT	Glycerol dialkyl glycerol tetraethers
GTGT	Glycerol trialkyl glycerol tetraethers
GMGT	Glycerol monoalkyl glycerol tetraethers
B&D	Bligh and Dyer
PI	Phosphatidyl inositol
PHexNAc	Phosphatidyl N-acetylhexosamine
PHexHex	Glycosylated phosphatidyl hexose
PG	Phosphatidyl glycerol
FAB	Fast atom bombardment
MALDI-TOF	Matrix-assisted laser desorption ionization-time of flight
